# Potentially Modifiable Micro-Environmental and Co-Morbid Factors Associated with Severe Wasting and Stunting in Children below 3 Years of Age in Aligarh District

**DOI:** 10.4103/0970-0218.66889

**Published:** 2010-04

**Authors:** Sandeep Sachdeva, Ali Amir, Mohd. A Ansari, Najam Khalique, Zulfia Khan, Seema Alam

**Affiliations:** Department of Community Medicine, Jawaharlal Nehru Medical College (JNMC), Aligarh Muslim University (AMU), Aligarh, Uttar Pradesh, India; 1 Department of Pediatrics, Jawaharlal Nehru Medical College (JNMC), Aligarh Muslim University (AMU), Aligarh, Uttar Pradesh, India

## Introduction

Undernutrition among children is a major public health problem in developing countries like India. The most commonly cited causative factors are food availability and dietary intake, breastfeeding, prevalence of infectious and parasitic diseases, access to health care, immunization against major childhood diseases, vitamin A supplementation, maternal care during pregnancy, water supply and sanitation, socio-economic status, and health-seeking behavior.(1) Children, especially the infants and toddlers, constitute the most disadvantaged group. The present study attempts to investigate the potentially modifiable distal and proximal factors that cause severe malnutrition in children under three years of age and suggests ways to mould them to their advantage.

## Materials and Methods

This community-based cross-sectional study was conducted among children under three years of age in the field practice areas of the Department of Community Medicine, J N Medical College, Aligarh. The estimated sample size was 468. A preformed questionnaire was administered carrying information about the socio-demographic profile and general physical examination of the child including signs of PEM and micronutrient deficiencies. Age and sex specific - 2 z-scores were followed to define wasting and stunting(CDC 2000 norms). WHO recommendations in terms of amount and frequency of complementary feeding for infants and children were used to categorize feeding as appropriate/not appropriate.(2)

## Results

Social class (odd’s ratio 2.3, 95% CI 1.0 to 5.1) and feeding practices (odd’s ratio 2.6, 95% CI 1.3 to 5.1) were the significant risk factors associated with wasting. However, only rural children were more likely to be stunted (odd’s ratio 2.1, 95% CI 1.3 to 3.1) and none of the other socio-demographic factors had significant association with stunting [Table 1]. Among the childhood morbidities, presence of measles (odd’s ratio 3.9, 95% CI 1.4 to 11.3), vitamin A deficiency (odd’s ratio 3, 95% CI 1.2 to 7.8), vitamin D deficiency (odd’s ratio 6.7, 95% CI 2.5 to 17.8) and worm infestation (odd’s ratio 3.3, 95% CI 1 to 10.3) were significantly associated with wasting. Pallor (odd’s ratio 129.7, 95% CI 54.2 to 310.8) was found to be the most significantly associated factor with stunting. Another factor that had a borderline association with stunting was the presence of vitamin D deficiency (odd’s ratio 3.01, 95% CI 1.3 to 7.2) [Table 2]. On binary logistic regression analysis, social class (adjusted OR 1.7, 95% CI 1.1 to 2.6), feeding practices (adjusted OR 2.8, 95%CI 1.4 to 5.9), Measles (adjusted OR 5.2, 95%CI 1.4 to 19.3), vitamin D deficiency (adjusted OR 5.3, 95%CI 1.6 to 17.9) were the factors found to be significantly associated with wasting. Only pallor (adjusted OR 1.7, 95%CI 1.1 to 2.6) was found to be significantly associated with stunting.

**Table 1:** Univariate analysis of the demographic and feeding related determinants of severe wasting and stunting

Variable	Wasting			Stunting		
	Severe	Not severe	OR (95% CI)	Severe	Not severe	OR (95% CI)
Locality						
Urban	6	135	0.4 (0.2 to 1.1)	57	84	2.1(1.3 to 3.1)
Rural	30	297		82	245	
Family Size						
≤6	19	172	1.28 (0.9 to 1.9)	51	140	0.6 (0.3 to 0.2)
>6	17	260		88	189	
Social class						
Lower	28	263	2.3 (1.0 to 5.1)	89	202	1.1 (0.7 to 1.1)
Upper	8	169		50	127	
Feeding						
Appropriate	19	131	2.6 (1.3 to 5.1)	92	226	1.1 (0.7 to 1.7)
Inappropriate	17	301		47	103	
Family						
Nuclear	24	266	1.3 (0.6 to2.6)	77	213	0.7 (0.5to 1.1)
Joint	12	166		62	116	
Dwellings						
Crowded	28	360	1.4 (0.6 to 3.3)	118	270	0.8 (0.5 to 1.4)
Not crowded	8	72		21	59	
Drainage						
Open	32	354	1.8 (0.6 to 5.1)	116	270	1.1 (0.7 to 1.9)
Closed	4	78		23	59	
Maternal age						
≤20	8	146	1.8 (0.8 to 4.1)	49	105	1.1 (0.8 to 1.5)
>20	28	286		90	224	

**Table 2:** Univariate analysis of child morbidity related determinants of severe wasting and stunting

Variable	Wasting			Stunting		
	Severe	Not severe	OR (95% CI)	Severe	Not severe	OR (95% CI)
Measles						
Present	5	17	3.9 (1.4 to 11.3)	8	14	1.4 (0.6 to 3.4)
Absent	31	415		131	315	
ARI*						
Present	2	17	1.4 (0.3 to 6.5)	9	10	2.21 (0.9 to 5.6)
Absent	34	415		130	319	
Pallor						
Present	17	164	1.5 (0.7 to 2.9)	133	48	129.8 (54.2 to 310.8)
Absent	19	268		6	281	
Vitamin A deficiency						
Present	6	30	3 (1.2 to 7.8)	13	20	1.6 (0.8 to 3.3)
Absent	27	405		126	309	
Vitamin D deficiency						
Present	7	15	6.7 (2.5 to 17.8)	12	10	3.01 (1.3 to 7.2)
Absent	29	417		127	319	
Diarrhoea*						
Present	3	20	1.9 (0.5 to 6.6)	10	13	1.9 (0.8 to 4.4)
Absent	33	412		129	316	
Worms						
Yes	4	16	3.3 (1 to 10.3)	9	11	2 (0.8 to 4.9)
No	32	416		130	318	

* Had one or more episodes in the past one month

## Discussion

Children belonging to the lower social classes and where feeding was inappropriate were significantly more wasted than the rest. These results were confirmed by other workers(3) indicating that unavailability of food, insufficient purchasing power, inappropriate distribution and inadequate utilization might make the children vulnerable to malnutrition in a deprived community. Long duration of breastfeeding without introduction of appropriate complementary feeds may be associated with higher malnutrition because it reflects lack of resources to provide children with adequate nutrition.(4) It is also possible that children who are breastfed for a long time are more reluctant to eat other foods, as was found in a study on a cohort of Ghanaian children.(5) Moreover, the present study found measles an important co-morbidity associated with severe wasting which has been seen in a case control study of severely malnourished children with diarrhoea in Bangladesh.(6) Whereas a Ugandan study found presence of fever in the preceding two week period as a risk factor for wasting.(7) Surprisingly, we also found vitamin D deficiency significantly associated with wasting. This could be due to wasting and stunting present together in some children. In contrast to the present study which only found pallor as a risk factor for severe stunting, some workers from the developing world have found family size, parental education, poor breastfeeding pattern and inadequate complimentary feeding associated with chronic malnutrition.(7–9) In a study done in Kerala anemia was seen to be significantly associated with mild and moderate malnutrition.(10)

There is a close positive link between the nutritional status of pre-school children and the stages of development of the states. Mothers’ education and household conditions have important influences on children’s health status irrespective of the stage of development.(11) Uttar Pradesh, where the present study was carried out, is one of the worst affected states in India. Therefore, focus should be on domiciliary management of severely malnourished children. Intervention programs for the most vulnerable groups should be planned to mitigate childhood morbidity and mortality. Micronutrient supplementation and health education of the caregivers through simple health packages would go a long way in alleviating the co-morbidities.
